# Enhancing strength and sustainability of concrete with steel slag aggregate

**DOI:** 10.1038/s41598-025-01296-5

**Published:** 2025-05-16

**Authors:** Ibrahim Kamal Ibrahim, Mohammed Rady, Nouran M. Tawfik, Mahmoud Kassem, Sameh Youssef Mahfouz

**Affiliations:** 1 Construction and Building Engineering Department, College of Engineering and Technology, Arab Academy for Science, Technology and Maritime Transport (AASTMT), B 2401 Smart Village, Giza, 12577 Egypt; 2 Civil & Construction Engineering, School of Engineering & Applied Science, Nile University, Giza, Egypt; 3 Smart Engineering Research Centre, SESC, Nile University, Giza, Egypt; 4 Construction and Building Engineering Department, College of Engineering and Technology, Arab Academy for Science, Technology and Maritime Transport (AASTMT), P.O. Box 2033, Elhorria, Cairo Egypt

**Keywords:** Sustainability, Green concrete, Industrial waste aggregates, Mechanical properties, X-ray diffraction, Differential thermal analysis, Civil engineering, Engineering

## Abstract

This study investigates the transformation of steel slag, a by-product of the steel industry, into a sustainable construction material by substituting it for natural aggregates in concrete mixtures. To this end, we have conducted an experimental program that evaluated 28 mix designs, along with a control mix based on natural aggregates, cement, silica fume, and chemical admixtures, to assess the effects of replacing natural aggregates with steel slag. Some mixes also incorporated different fiber types to enhance performance. The study assessed the mechanical and physical properties of both fresh and hardened concrete to evaluate the overall effects of these alterations. Furthermore, thermogravimetry-differential thermal analysis (TG-DTA) and X-ray diffraction (XRD) were employed to characterize the mineralogical composition and thermal behavior of the concrete mixes. To further validate the compressive strength improvements and quantify data variability, a statistical analysis was conducted. The results revealed that concrete mixtures utilizing steel slag demonstrated substantial enhancements in compressive strength, with four mixtures featuring complete replacement of natural aggregates achieving more than 1.7 times the strength of control mix. The inclusion of fibers further enhanced performance in terms of crack resistance and post-cracking behavior. The TG-DTA and XRD analyses revealed that the steel slag promotes additional hydration and the formation of calcium silicate hydrate, resulting in a denser microstructure. Furthermore, the statistical analyses confirmed that these improvements are statistically significant, highlighting the potential of steel slag and fiber reinforcement to enhance both the fresh and hardened properties of concrete. The current study demonstrated that steel slag can fully replace natural aggregates while enhancing concrete performance, offering a sustainable solution for both construction material sourcing and industrial waste management.

## Introduction

Concrete has been extensively utilized in the construction industry due to its versatility^1,2^. Due to the continuous growth in building demands, the industry generates large waste and industrial by-products such as steel slag (SS). Thus, the construction sector has developed strategies to decrease waste and maximize recycling in response to rising regulatory pressures to implement the circular economy concept^3–6^. Utilizing various industrial wastes has several benefits, such as avoiding disposal expenses, mitigating soil and air pollution, saving natural resources, and reducing the costs of construction materials.

The global steel industry produces vast quantities of SS waste. SS, a byproduct constituting approximately 15–20% of crude steel production by mass, is generated globally in substantial quantities, about 7 million tons annually (projected to rise to 12 million tons by 2025 due to increased construction demand), with India producing 4 million tons per year (expected to reach 60 million tons by 2030), China discarding over 80 million tons annually with only a 22% recovery rate, and Europe generating around 20 million tons per year^7^. This by-product poses significant environmental challenges, including the potential leaching of heavy metals such as lead, zinc, and chromium when not properly stabilized^8^, leading to high disposal costs, estimated between 20 and 50 USD per ton. Given these challenges, the sustainable management of SS has become an essential research topic.

Numerous studies have examined the impact of SS on concrete mixtures, exploring its potential to enhance performance and sustainability. For instance, Ho et al.^9^ investigated the use of SS as a full replacement for natural coarse aggregates in high-performance concrete (HPC), coupled with partial cement substitution by fly ash (FA) and ground granulated blast-furnace slag (GGBFS). Eventually, they demonstrated that HPC containing 100% SS exhibited a 20–21% higher unit weight than conventional concrete. Moreover, the addition of FA and GGBFS further enhanced the mechanical strength, reduced drying shrinkage, and lowered both material cost and environmental impacts by mitigating global warming and ozone depletion potentials.

Mu et al.^10^ explored the production of clinker-free ultra-high-performance concrete (UHPC) using a solid waste-based binder and SS aggregates. Through an orthogonal test design, they optimized critical parameters, refining slag content, gypsum content, GGBFS–hot stifle steel slag ratio, and binder–sand ratio, to achieve compressive and flexural strengths of 108.39 MPa and 37.22 MPa, respectively. Microanalytical techniques, including X-ray diffraction (XRD), Fourier-transform infrared spectroscopy (FT-IR), thermogravimetry-differential scanning calorimetry (TG-DSC), and scanning electron microscopy (SEM-EDS), revealed that the hydration products were ettringite and C–(A)–S–H gels, formed through synergistic reactions, resulting in a denser, more compact microstructure. Biskri et al.^11^ compared HPC produced with artificial aggregates, specifically SS and crystallized slag to HPC made with natural limestone aggregates. Maintaining a constant water-to-binder ratio of 0.27 and supplementing the mixtures with GGBFS and silica fume, they found that the mineralogy, morphology, and strength of the aggregates significantly influence both compressive strength and durability. The rough surface texture of artificial aggregates enhanced the bond between the aggregates and the hardened cement paste, leading to improved performance.

In another approach, Andrade et al.^12^ evaluated the potential of using SS as an alternative aggregate in eco-friendly structural concretes. By fully replacing conventional aggregates with SS and employing a PCE-based superplasticizer, their accelerated carbonation tests revealed that SS concretes achieved higher compressive strengths and exhibited up to a 60% reduction in carbonation depth compared to traditional mixes. Similarly, Afroughsabet et al.^13^ produced high-performance steel fiber-reinforced concrete using recycled concrete aggregates (RCA) to replace natural aggregates. In their study, RCA derived from parent concretes with varying strengths (40 and 80 MPa) was used at replacement ratios of 50% and 100%, along with 1% double hooked-end steel fibers and, in some mixtures, a 30% substitution of Portland cement with slag. Their results confirmed that high-quality RCA, particularly when combined with slag and steel fibers, can yield concrete with improved mechanical properties and enhanced durability indicators such as reduced water absorption and shrinkage.

Le et al.^14^ contributed to the development of smart UHPCs by investigating the self-sensing behavior of concrete incorporating SS aggregates and steel fibers. By replacing traditional silica sands with SS aggregates and varying the particle size, SS-to-cement ratio, and steel fiber content, they found that an optimal mix, with SS aggregates of 0.39 mm maximum diameter, an SS-to-cement ratio of 0.5, and 2 vol% steel fibers, produced the highest fractional change in resistance (42.9%) and stress sensitivity (0.298%MPa). These results highlight the importance of filler distribution in enhancing the self-sensing capabilities of UHPC. Rehman et al.^15^ examined the combined effect of glass powder (GP) and granular steel slag (GSS) as partial replacements for cement and fine aggregates in self-compacting concrete (SCC). Their investigation demonstrated that increasing GP content improved workability, while higher GSS content slightly reduced it. Nevertheless, mechanical properties such as compressive strength, splitting tensile strength, flexural strength, and modulus of elasticity improved significantly, with maximum increases reaching 11%, 13.2%, 19.3%, and 20%, respectively, when compared to conventional mixes.

Zhou et al.^16^ focused on the long-term stability of SS sand in mortar and concrete by comparing rapid test methods with extended alkaline-aggregate reaction tests conducted over periods up to 156 weeks. Their findings indicated that rapid tests tend to exaggerate the hazards associated with SS sand, whereas long-term tests provide a more accurate assessment of stability. They reported that while the replacement ratio of SS sand minimally affects volumetric changes, surface damage, especially in samples with particle sizes larger than 1.18 mm, is significantly linked to the uneven distribution of free CaO. Tang et al.^17^ addressed the reactivity concerns of SS by investigating carbonation treatment and the removal of fine particles (< 75 μm) to enhance slag stability. They evaluated four variants of SS (raw, raw classified, carbonated, and carbonated classified) as replacements for natural fine aggregates in nine concrete and mortar mixes. Their results showed that carbonated classified SS significantly reduced mortar shrinkage, alkali-silica reaction, and water absorption while enhancing compressive and flexural strengths, particularly when used at a 50% replacement level.

Santamaría et al.^18^ assessed the durability of structural concrete mixes made with electric steelmaking aggregates under both normal and aggressive conditions. Their study demonstrated that pumpable and self-compacting concrete containing these aggregates exhibit excellent physical, mechanical, and dimensional stability, even after rigorous freeze-thaw, drying-wetting, tidal immersion, and reinforcement corrosion tests. This confirms the suitability of these concretes for a variety of structural applications. Costa et al.^19^ evaluated the durability of concrete incorporating SS as both an aggregate and a mineral admixture (SS powder), with a focus on resistance to chloride attack. Compared to reference concretes using conventional aggregates and commercial mineral admixtures (silica fume and metakaolin), the SS concretes exhibited lower chloride penetration depths, reduced water absorption, and enhanced compressive and tensile strengths. Moreover, the SS powder demonstrated a capacity similar to silica fume in forming Friedel salt, further contributing to the improved durability.

**Fig. 1:**
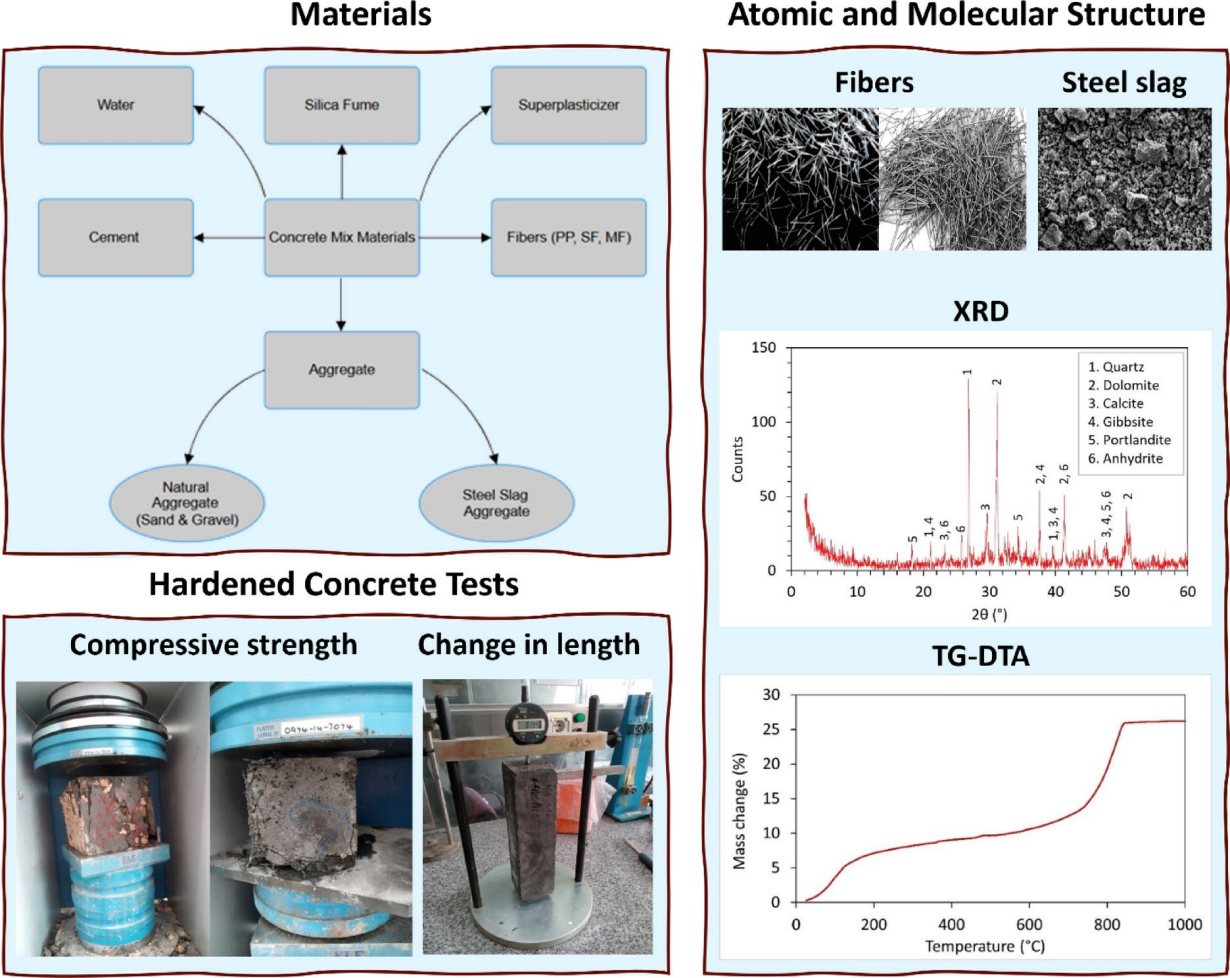
Summary of the methods and tests conducted in the present study,.

Despite the efforts of previous studies to assess the utilization of steel slag in concrete, most studies focused on replacing natural coarse aggregates with steel slag; only a few have explored its potential as a fine aggregate replacement, and even fewer have investigated the effects of incorporating various fiber types. To address this gap, our study examines the use of steel slag as a replacement for both coarse and fine aggregates. Moreover, we incorporate various types of fibers, including polypropylene, steel, and macro fibers, to investigate their effects with steel slag aggregates in concrete mixes. To this end, twenty-nine concrete mix designs, including a control mix and mixes with varying steel slag proportions and fiber contents, were systematically evaluated through extensive experimental testing. Eventually, we assessed mechanical properties (such as compressive strength at 7 and 28 days), durability, microstructural characteristics (via TG-DTA and XRD analyses), and performed statistical analyses to validate our findings. Figure 1 illustrates the overall materials and tests conducted in the present study.

## Materials

**Fig. 2:**
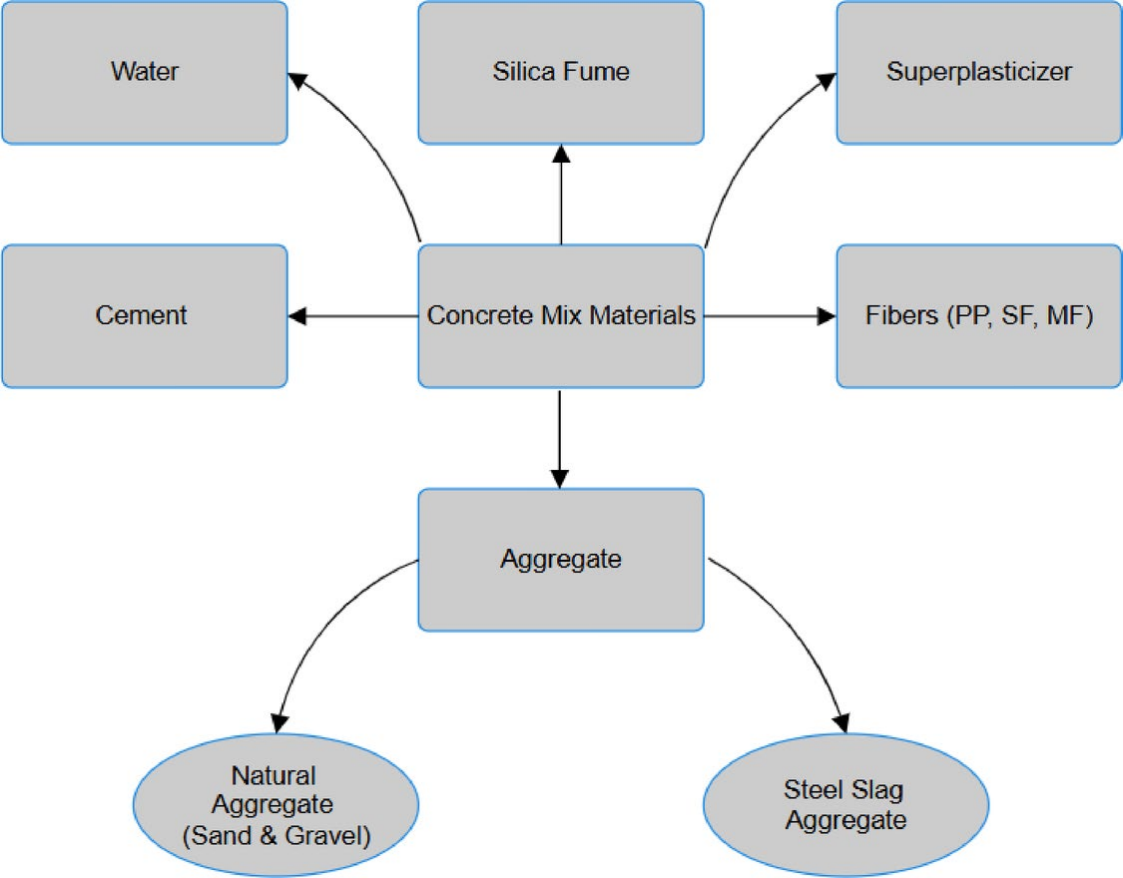
Utilized materials in the present study.

Figure 2 shows the materials utilized in the present study. Laboratory experiments were conducted to assess the physical and mechanical properties of the materials used. The material specimens are prepared and tested according to the requirements of the American society for testing and materials (ASTM), the Egyptian code of practice (ECP), and Egyptian standard specifications (ESS).

The Ordinary Portland cement (OPC Type I) is a binding material, primarily composed of clinkers and various other constituents. Table 1 defines the chemical properties of the OPC utilized in this work. Samples of OPC were experimentally tested to assure the compliance of cement to ASTM C150/C150M-12^20^ and ESS 4756-1^21^. The test methods illustrated in ASTM C786/C786M-10^22^, ASTM C191-13^23^, and ESS 2421^24^ for determining the cement fineness and paste setting times were utilized. A 150-µm (No. 100) sieve determined a cement fineness of 97.2%, with the initial and final setting times measured at 64 and 307 min (compared to allowable limits of 45 and 375 min, respectively). Potable, fresh water, free from impurities, was used to ensure optimal hardening, strength, and durability in accordance with ECP 203–2020^25^.

**Table 1:** Chemical composition of OPC.

Property	Content (%)
SiO_2_	20.85
Al_2_O_3_	4.23
FeO_3_	5.25
CaO	63.49
Na_2_O	0.16
K_2_O	0.4
SO_3_	2.38
Insoluble residue	0.18
Cao free	1.16
Available alkali (Na_2_O equivalent)	0.42
Loss on ignition	1.05

The fine and coarse natural aggregates used were siliceous sand and crushed dolomite rocks, respectively. Both coarse and fine aggregates were clean and free from impurities. As per ASTM C127-15^26^and ASTM C128-15^27^, the specific gravity and water absorption test methods were carried out for coarse and fine aggregates, respectively. The specific gravities were 2.605 g/cm^3^ and 2.651 g/cm^3^ for coarse and fine aggregates, respectively. The water absorption percentages were 3.03% ad 1.18% fr coarse and fine aggregates, respectively. High-range water reducers (HRWR) superplasticizers were utilized to reach suitable workability. The HRWR reduces water content by 12–30% and can be added to a mixture with a low-to-normal slump and water-cement ratio to make the high-slump flow. These superplasticizers complied with the standards of the chemical additives (ASTM C494 Type G^28^ and ESS 1899-1^29^). Silica fume was used as a pozzolanic additive to improve the mechanical properties of concrete. The silica fume particle is approximately 100 times smaller than the average cement particle. The small size of silica fume particles allows them to penetrate between mortar grains (i.e., cement and sand), reducing the water-cement ratio. The chemical composition of the utilized silica fume conformed to ASTM C1240-14^30^ and ESS 5129-1^31^. Table 2 displays the chemical analysis and physical features of the silica fume utilized in this study.

**Table 2:** Chemical analysis and physical properties of the silica fume used.

Property	Content (%)
SiO_2_	90.2
Fe_2_O_3_	0.4
Al_2_O_3_	1.7
MgO	1.7
CaO	2.1
SO_3_	0.5
K_2_O	0.7
Na_2_O	0.7
Density	~ 0.65 kg/L
Total Chloride Ion Content	< 0.3 M-%

**Fig. 3:**
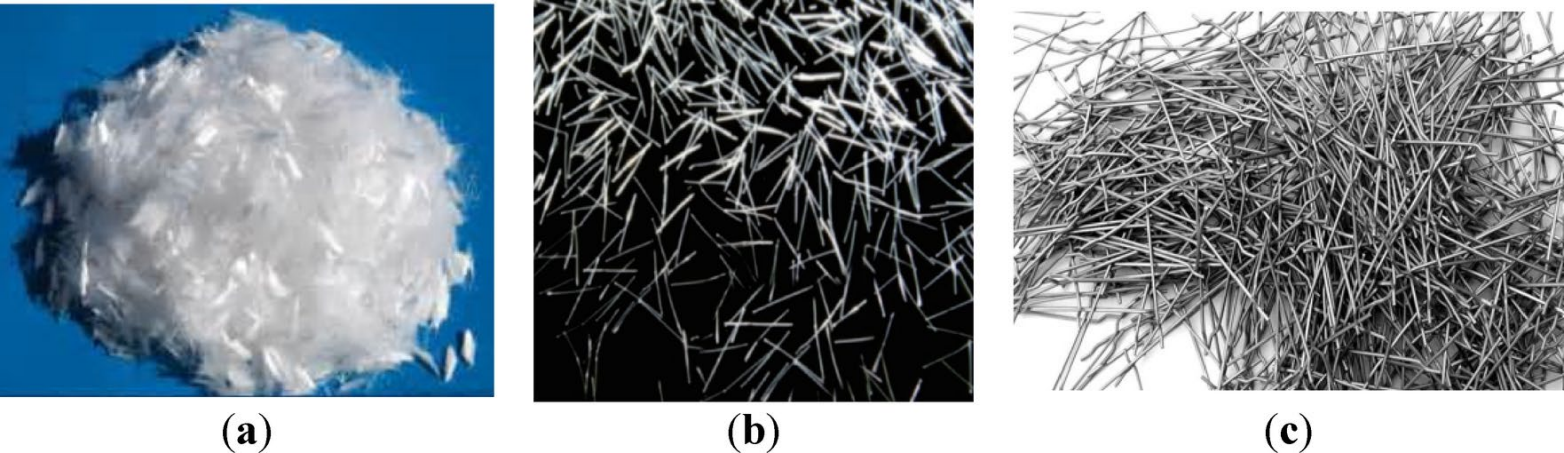
SEM micrograph for different fiber types: (**a**) PP (**b**) MF (**c**) SF.

Three fiber types, polypropylene (PP), macro fibers (MF), and steel fibers (SF) were integrated into the mix design of chosen concrete mixtures. Figure 3 displays the SEM micrograph depicting the different fiber types employed in this study. PP fibers are made of 100% virgin homopolymer polypropylene fibrillated fibers. They were used to mitigate the development of internal cracks and resist the impact forces, shattering forces, and material losses due to abrasion and water migration. MFs were designed to minimize the development of concrete fracturing plastic shrinkage and enhance the resistance of toughness, impact, and fatigue. Hooked-end SF was used to enhance the mechanical properties of concrete, such as flexural strength, compressive strength, and dynamic fracture properties. Tables 3 and 4, and Table 5 summarize the primary characteristics of PP, MF, and SF, respectively.

**Table 3:** Main properties of polypropylene fibers.

Property	Value
Length (L)	12 mm
Diameter (D)	0.05 mm
Aspect ratio (L/D)	240
Specific gravity	0.91
Tensile strength	Approx. 350 MPa
Melting point	Approx.165 ^o^C
Resistance to acids/alkalis	Excellent
Color	Transparent
Modulus of elasticity	Approx.1000 MPa
Constituent	Pure homopolymer Polypropylene

**Table 4:** Main properties of macro fibers.

Property	Value
Length (L)	48 mm
Compact density	0.91 kg/dm^3^
Tensile strength	> 500 MPa
Melting point	280 ^o^C
Diameter (D)	0.9 mm
Aspect ratio (L/D)	53.33
Chemical resistance	High
Color	Black fiber
Modulus of elasticity	> 20 GPa
Constituent	100% polyolefin

**Table 5:** Main properties of End-Hooked steel fibers.

Property	Value
Size	60 mm x 0.9 mm
Length (L)	60 mm (+/−3 mm)
Diameter (D)	0.9 mm (+/−0.05 mm)
Aspect ratio (L/D)	66.67
Middle length	40 mm (+/−3 mm)
Height of hook (HH)	(2.10–2.90) mm
Length of hook (HL)	(4–6) mm
Tensile strength	Minimum 1000 MPa

**Fig. 4:**
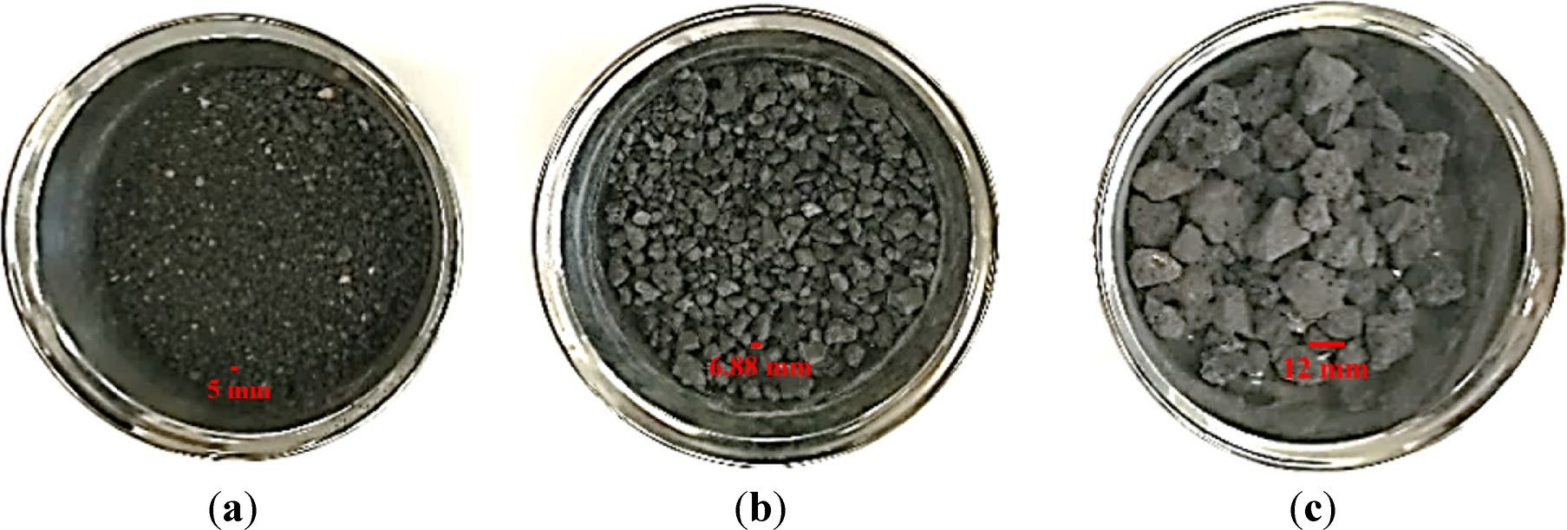
Different steel slag sizes used in this study with scale bars: (**a**) fine steel slag passing through a 4.75 mm sieve (particle size < 4.75 mm); (**b**) coarse steel slag retained on a 4.75 mm sieve (4.75–9.5 mm); (**c**) coarse steel slag retained on a 9.5 mm sieve (> 9.5 mm).

**Fig. 5:**
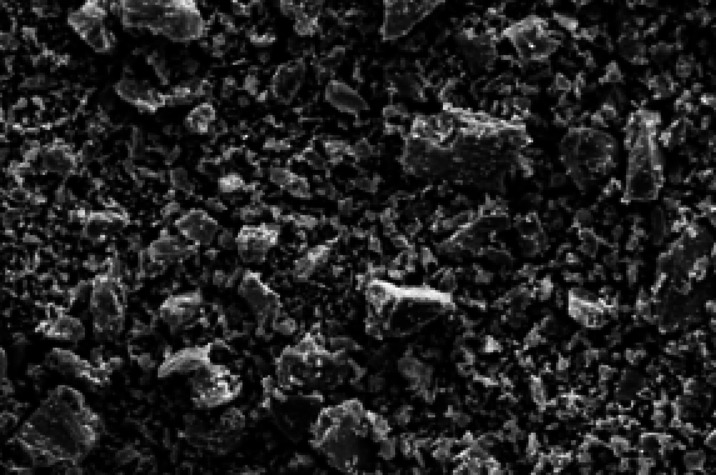
SEM micrograph for steel slag.

Three classes of grain steel slag are employed: those retained on a 4.75 mm filter, those passing through a 4.75 mm screen, and those retained on a 9.50 mm sieve, as seen in Fig. 4. Figure 5 displays the SEM micrograph of the steel slag. Tables 6 and 7 present the chemical composition and physical properties of the steel slag employed in this study, respectively. The steel slag utilized in this study was obtained from Egypt Stone for Mining and Supplies.

**Table 6:** Chemical composition of steel slag.

Property	Content (%)
SiO_2_	29.30
Al_2_O_3_	10.80
Fe_2_O_3_	21.50
CaO	29.10
MgO	5.32
SO_3_	0.35
Na_2_O	0.49
TiO_2_	0.45
P_2_O_5_	0.63
Cr_2_O_3_	0.41
MnO	0.97
Cl-	0.08
Loss on ignition	0.32

**Table 7:** Physical properties of the steel slag.

Property	Value
Apparent specific gravity	3.46
Dry specific gravity	3.42
Saturated specific gravity	3.43
Bulk density (ton/m^3^)	1.98
Water Absorption (%)	0.3

**Fig. 6:**
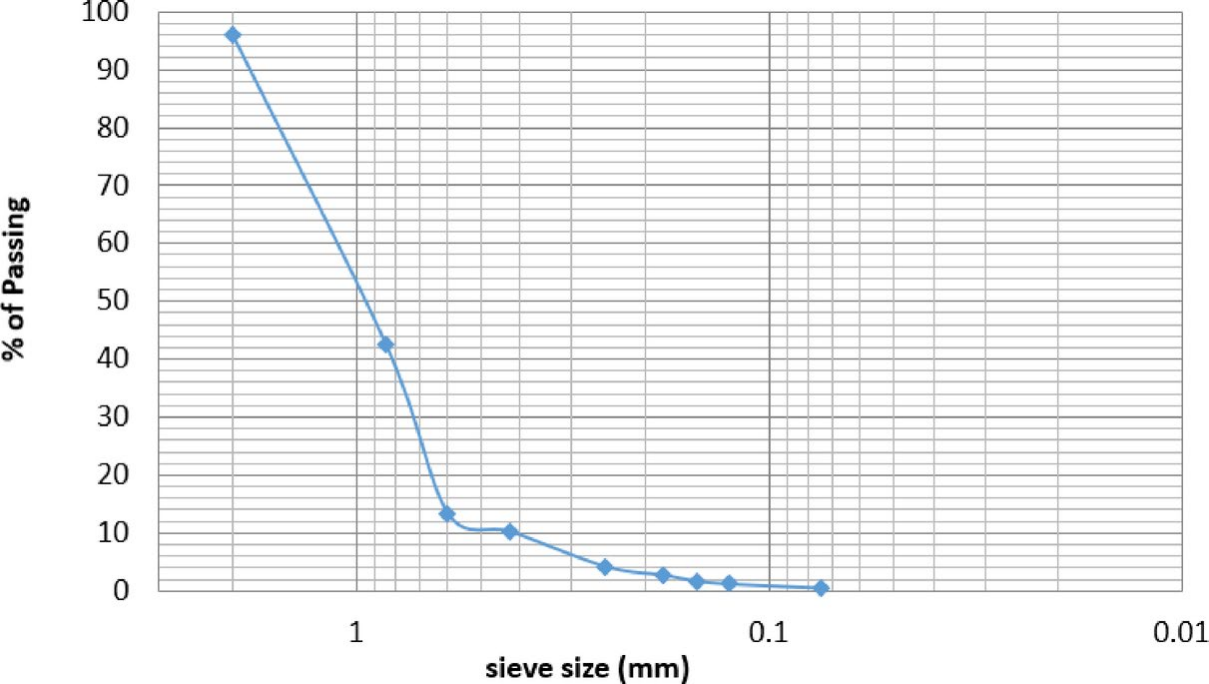
Cumulative percent passing versus the logarithmic sieve size.

The sieve analysis of the steel slag samples revealed that the material comprises a mixture of fine and coarse aggregates. The Sieve analysis is compared to the relevant limit as per ASTM C136-06^32^. The findings of the sieve analysis are presented Table 8; Fig. 6.

**Table 8:** Grading of utilized steel slag.

Sieve (mm)	Retained	Cumulative	% Retained	% Passing
20	0.5	0.5	5.025	94.975
14	1.9	2.4	24.121	75.879
10	0.8	3.2	32.161	67.839
5	1.2	4.4	44.221	55.779
2.36	0.2	4.6	46.231	53.769
1.18	0.2	4.8	48.241	51.759
0.6	4.0	8.8	88.442	11.558
0.3	0.6	9.4	94.472	5.528
0.15	0.5	9.9	99.497	0.503
Pan	0.05	9.95	100	ــ

## Experimental program

The mix design in this study was developed to achieve a characteristic strength of 60 MPa, ensuring a high-performance concrete mix. The absolute volume method, as recommended by ECP 203–2020 and ACI committee 211, was utilized to determine the material proportions for each cubic meter of concrete, ensuring a balanced and optimized mix. The control mix was designed using Ordinary Portland Cement (OPC), silica fume, water, fine aggregate (sand), coarse aggregate (dolomite), and a superplasticizer to enhance workability. The cement content (475 kg/m³) and silica fume (75 kg/m³) were selected to enhance strength and durability, while the water-to-binder ratio (w/b = 0.35) was chosen to ensure high strength while maintaining adequate workability. The mix comprised 520 kg of fine aggregate and 1040 kg of coarse aggregate. The superplasticizer (8.25 kg/m³) was incorporated to improve flowability and reduce water demand, preventing excessive porosity. For the twenty-eight subsequent mixes, natural aggregates were partially or fully replaced with steel slag aggregates of varying sizes to study their effect on strength and durability. The selection of slag particle sizes (retained on 9.5 mm sieve, retained on 4.75 mm sieve, and passing 4.75 mm sieve) was based on their influence on packing density, interlocking, and hydration enhancement. Additionally, fiber reinforcement (polypropylene, macro fibers, and steel fibers) was incorporated into selected mixes to assess their impact on crack resistance, tensile strength, and ductility. The fiber dosage was carefully determined based on past studies and industry recommendations, ensuring improved mechanical properties without compromising workability, with fiber volume fractions for PP, MF, and SF equal to 0.12%, 0.68%, and 0.39%, respectively. The weights of OPC, silica fume, water, and superplasticizer were constant for all mixes. Table 9 presents the compositions of concrete constituents per cubic meter for different mixes.

**Table 9:** Proportions of concrete mixes. (PP: polypropylene fiber**s;** SF: Hooked-end steel fiber**s;** MF: macro fibers).

Mix.	Sand (kg)	Gravel (kg)	Cement (kg)	Water (kg)	Silica fume (kg)	Fine steel slag passing from 4.75 mm sieve (kg)	Coarse steel slag retained on 4.75 mm sieve (kg)	Coarse Steel slag retained on 9.50 mm sieve (kg)	Superplasticizer (kg)	Fibers (kg)
1	520.00	1040.00	475.00	186.00	75.00	-	-	-	8.25	-
2	-	-	475.00	186.00	75.00	-	-	1367.00	8.25	-
3	-	-	475.00	186.00	75.00	-	1367.00	-	8.25	-
4	-	-	475.00	186.00	75.00	1367.00	-	-	8.25	-
5	-	-	475.00	186.00	75.00	-	456.00	912.00	8.25	-
6	-	-	475.00	186.00	75.00	456.00	-	912.00	8.25	-
7	-	-	475.00	186.00	75.00	456.00	912.00	-	8.25	-
8	-	987.20	475.00	186.00	75.00	493.62	-	-	8.25	-
9	-	987.20	475.00	186.00	75.00	-	493.62	-	8.25	-
10	433.40	866.80	475.00	186.00	75.00	216.70	-	-	8.25	-
11	293.70	587.40	475.00	186.00	75.00	587.40	-	-	8.25	-
12	433.40	866.80	475.00	186.00	75.00	-	216.71	-	8.25	-
13	293.70	587.40	475.00	186.00	75.00	-	587.44	-	8.25	-
14	473.80	-	475.00	186.00	75.00	-	-	947.75	8.25	-
15	374.10	748.20	475.00	186.00	75.00	-	-	374.11	8.25	-
16	205.40	410.80	475.00	186.00	75.00	-	-	821.75	8.25	-
17	374.80	-	475.00	186.00	75.00	-	947.75	-	8.25	-
18	374.10	748.20	475.00	186.00	75.00	-	374.11	-	8.25	-
19	374.10	410.80	475.00	186.00	75.00	-	821.75	-	8.25	-
20	-	-	475.00	186.00	75.00	1367.00	-	-	8.25	PP (0.90)
21	-	-	475.00	186.00	75.00	1367.00	-	-	8.25	SF (25.00)
22	-	-	475.00	186.00	75.00	456.00	912.00	-	8.25	PP (0.90)
23	-	-	475.00	186.00	75.00	456.00	912.00	-	8.25	SF (25.00)
24	-	-	475.00	186.00	75.00	684.00	-	684.00	8.25	PP (0.90)
25	-	-	475.00	186.00	75.00	684.00	-	684.00	8.25	SF (25.00)
26	-	-	475.00	186.00	75.00	684.00	-	684.00	8.25	-
27	-	-	475.00	186.00	75.00	1367.00	-	-	8.25	MF (5.00)
28	-	-	475.00	186.00	75.00	456.00	912.00	-	8.25	MF (5.00)
29	-	-	475.00	186.00	75.00	684.00	-	684.00	8.25	MF (5.00)

### Concrete properties tests

The following tests were performed in the testing of materials research laboratory. These tests were carried out to determine the main properties of the concrete in the fresh and hardened stages.

#### Workability

Workability reflects the quality of freshly mixed concrete. A slump test was conducted as per ASTM C143^33^ to measure workability. A standard cone was positioned on a solid level and filled with fresh concrete in three equal layers. Each layer was compacted 25 times using a steel rod in a defined manner. Eventually, the cone was carefully removed, and the final height of the fresh concrete was subtracted from the initial cone height.

#### Unit weight

The unit weight depends on the initial concrete materials and changes in volume. The regulations specified by ASTM C138/C138M-17a^34^ were followed to calculate the unit weight of fresh concrete. The unit weight was obtained by the weight and volume quotient of the concrete sample.

#### Compressive strength

**Fig. 7:**
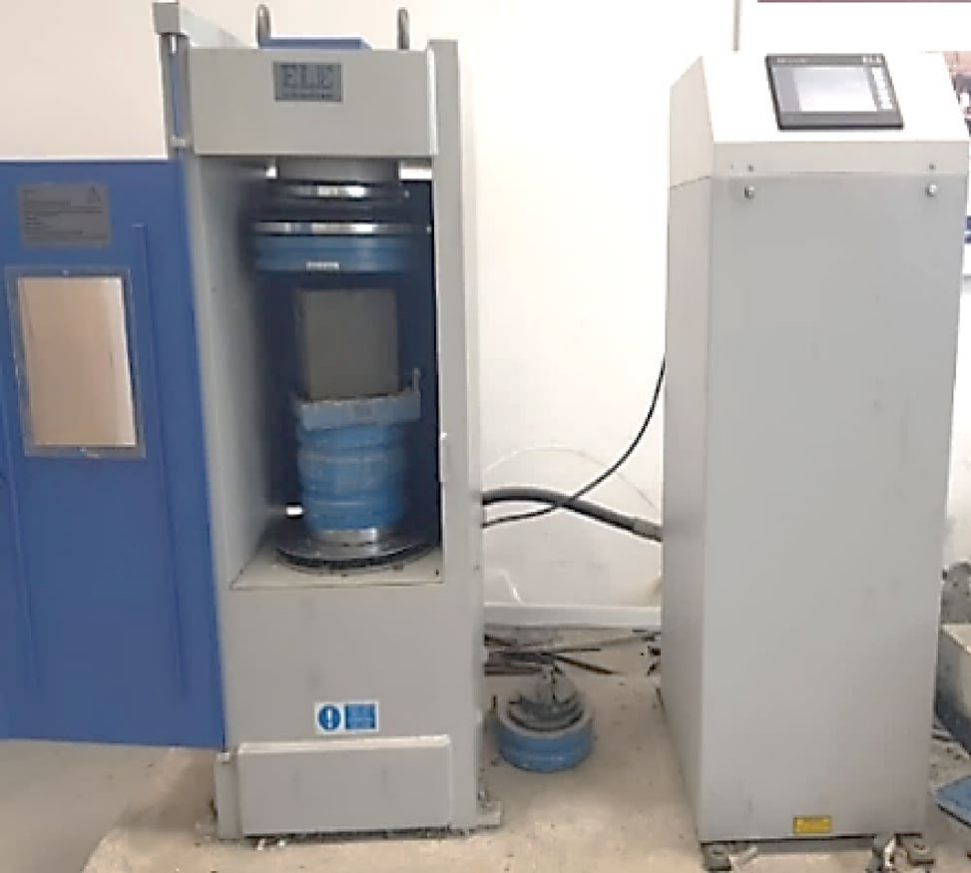
Compression testing machine.

The compressive strength was tested using the uniaxial compression test method as per ESS 1658^35^. We cast the specimens in standard steel molds with dimensions 150 mm × 150 mm × 150 mm. The cubes were left in dry conditions for 24 h, and then they were de-molded and cured in a clean water chamber as per ASTM C192/C192M-19^36^. Three cubes of each mixture are tested at 7 days, and three cubes at 28 days of age are tested using a 2000 kN automated compression testing machine (ADR Auto v2.0). Figure 7 shows a sample of a concrete mix during the compression test.

#### Change in length

**Fig. 8:**
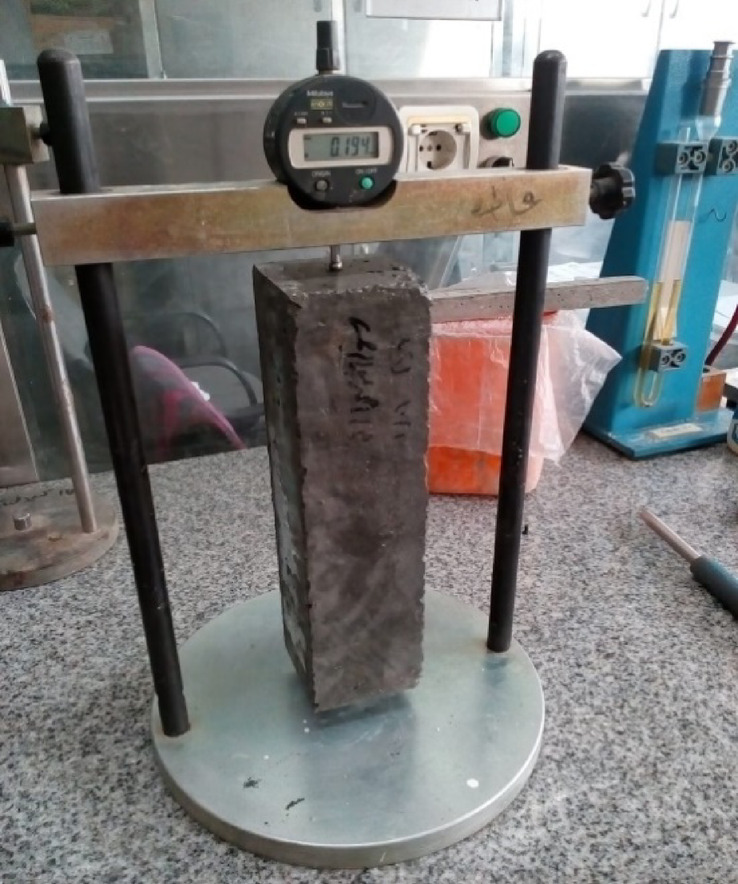
A concrete prism sample during the measurement of the length change.

The change in specimen length was measured for different mixes. Prisms with 75 × 75 × 285 mm dimensions were established for each mix. The initial lengths of specimens were measured 24 h after being removed from mold. The prisms were then put in a lime bath and examined after 1, 7, 14, and 28 days. Eventually, the difference in length was calculated using Eq. (1) as indicated by ASTM C157M-17^37^.

$$\:\varDelta\:{L}_{x}\:=\:\frac{CRD-initial\:CRD}{G}\:\times\:\:100\:\:\:\:\:\:\:\:\:\:\:\:\:\:\:\:\:\:\left(1\right)$$

where ΔL_x_ is the percentage change in specimen length at a particular age, CRD is the difference between the comparator reading of the specimen and the reference bar at a particular age, and G is the gage length. Figure 8 shows a concrete sample while the measurement of the length changes.

#### Atomic and molecular structure tests

X-ray diffraction (XRD) and simultaneous thermogravimetry and differential thermal analysis (TG-DTA) were performed to determine the atomic and molecular structure of different concrete mixes.

##### XRD test

XRD is a non-destructive method conducted to obtain information on the mixes’ crystallographic structures, physical characteristics, and chemical composition. The test was conducted utilizing an X’Pert-Pro X-ray diffractometer equipped with a monochromatic source of radiation (Cu-Kα). The scanning was employed within the range of $\:5^\circ\:\:\le\:2{\uptheta\:}\:\le\:60^\circ\:$ at a scan rate of 2°/minute.

##### TG-DTA test

In combination with differential thermal analysis (DTA), the specimens were placed in a ceramic cauldron and heated to 1000 °C at a consistent rate of 10 °C/min under the nitrogen atmosphere. The quantitative and qualitative investigations were completed by examining the mass loss of the paste at various temperatures.

## Results and discussions

The study was conducted by systematically preparing concrete mixes that were categorized into three groups to comprehensively evaluate the influence of steel slag on concrete performance. The first group comprises mixes where steel slag is used as a complete replacement for natural aggregates. The second group includes mixes with partial replacements of fine and coarse aggregates with steel slag, allowing us to assess the impact of different particle sizes and gradations. The third group consists of mixes where steel slag fully replaces natural aggregates and various types of fibers are incorporated, enabling an investigation into the combined effects of fiber reinforcement and slag properties. These categories were designed to study key parameters such as the effect of full versus partial steel slag replacement, the role of particle size distribution and gradation, and the impact of different fiber types on the compressive strength of concrete, while also correlating the observed changes in mineralogical composition and thermal behavior with strength variations. The results of the study are detailed in the subsequent sections, which are organized into discussions of fresh concrete properties, hardened concrete properties, and atomic and molecular structure properties.

### Fresh concrete properties

The challenges in achieving consistent workability with steel slag aggregates are attributed to their rough texture and irregular shape, which can reduce flowability. To address these issues, a High-Range Water Reducer (HRWR) superplasticizer was incorporated. The HRWR enabled a reduction in water content by 12–30%, which allowed to maintain a low-to-normal water-cement ratio while still producing a high-slump flow. As a result, the concrete mixtures achieved acceptable workability with slump values ranging from approximately 60 to 80 mm. The average unit weight of the concrete mixed with steel slag was around 2752 kg/m^3^.

### Hardened concrete properties

**Fig. 9:**
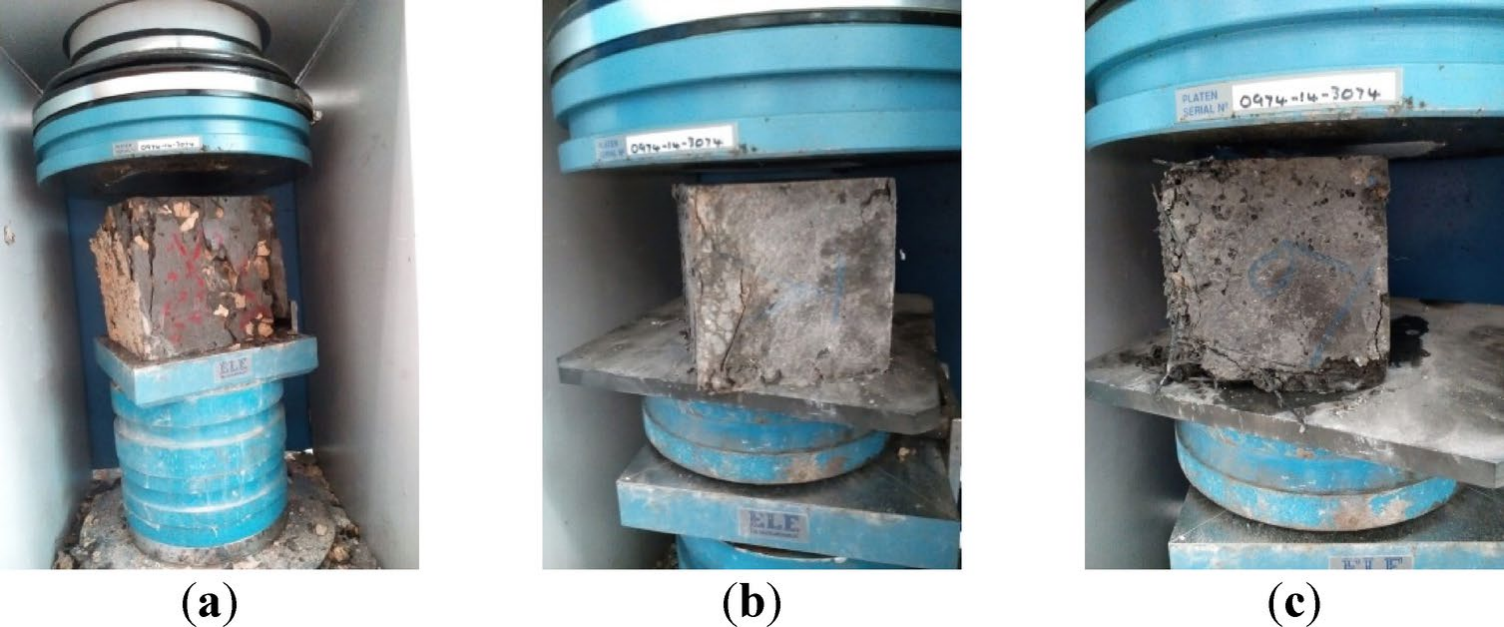
Concrete samples after the compression test: (**a**) control mix with natural aggregate (mix 1); (**b**) mix with fine and coarse steel slag aggregates (mix 6); mix with coarse steel slag aggregate and macro fibers (mix 29).

Figure 9 displays the failure patterns observed in selected concrete samples subjected to compressive loads, while Table 10 summarizes the compressive strength values for all concrete mixes tested at various ages. The compressive strength data clearly indicate that incorporating steel slag has had a beneficial effect on the concrete’s compressive strength. Steel slag, abundant in oxides such as CaO, SiO₂, Al₂O₃, and Fe₂O₃, as listed in Table 6, undergoes partial reaction with water to improve hydration. The higher CaO content facilitates the early production of Calcium Silicate Hydrate (C-S-H), while ongoing hydration enhances the strength and mechanical properties of concrete over time. Thus, the increase in the compressive strength can be attributed to the hydration effect of the steel slag. Moreover, factors such as the incorporation of fibres and the gradation of slag particle sizes further affect the strength and performance of concrete.

**Table 10:** Compressive strength results for the 7-day and 28-day for different concrete mixes.

Mix	Compressive strength (MPa)											
	7 days						28 Days					
	1	2	3	Mean	SD	% inc.	1	2	3	Mean	SD	% inc.
1	32.60	36.20	33.10	34.30	2.00	-	55.40	50.60	51.20	52.40	2.62	-
2	47.60	51.40	50.70	49.90	2.02	+ 46.91	63.70	54.60	60.0	59.43	4.57	+ 13.42
3	60.90	49.70	57.90	56.17	5.80	+ 65.36	79.10	78.40	77.40	78.63	0.95	+ 49.43
4	54.10	77.00	83.20	71.43	15.33	+ 110.30	The machine reaches its max limit				-	> 69.64
5	60.10	54.70	51.40	55.40	4.39	+ 63.10	80.80	79.50	71.60	77.30	4.98	+ 47.52
6	59.6	55.30	61.90	58.93	3.35	+ 73.50	86.80	79.80	88.90	85.17	4.77	+ 62.53
7	64.0	50.70	62.70	59.17	7.33	+ 74.09	The machine reaches its max limit				-	> 69.64
8	37.70	43.20	40.40	40.43	2.75	+ 19.04	60.00	64.90	60.70	61.87	2.65	+ 18.07
9	49.40	51.80	46.00	49.07	3.00	+ 44.46	59.50	52.40	51.20	54.37	4.49	+ 3.75
10	61.60	56.50	64.00	60.70	3.83	+ 78.70	77.00	83.10	80.10	80.07	3.05	+ 52.80
11	44.50	42.50	46.60	44.53	2.05	+ 31.11	70.40	68.20	67.40	68.67	1.55	+ 31.04
12	43.10	33.10	40.20	38.80	5.20	+ 14.23	51.60	52.40	59.50	54.50	4.35	+ 4.01
13	56.0	62.30	61.80	60.03	3.50	+ 76.74	80.10	83.10	71.80	78.33	5.85	+ 49.49
14	40.60	45.00	46.10	43.90	2.91	+ 29.24	61.70	70.30	67.20	66.40	4.41	+ 26.72
15	25.10	36.70	29.00	30.27	5.90	−10.89	47.80	42.40	48.10	46.10	3.21	−12.02
16	59.10	58.80	61.70	59.87	1.60	+ 76.25	72.20	76.80	71.80	73.60	2.78	+ 40.46
17	53.10	57.30	51.00	53.80	3.21	+ 58.39	68.60	69.90	64.90	67.80	2.60	+ 29.39
18	39.40	40.20	41.00	40.20	0.80	+ 18.35	56.50	51.40	55.30	54.40	2.67	+ 3.82
19	51.70	49.10	60.50	53.77	5.98	+ 58.29	63.20	68.60	61.70	64.50	3.63	+ 23.09
20	58.70	45.3	63.40	55.80	9.39	+ 64.28	72.20	59.90	88.80	73.63	14.51	+ 40.52
21	65.10	63.6	54.50	61.07	5.74	+ 79.78	97.10	84.60	70.80	84.17	13.16	+ 60.62
22	60.20	41.30	36.80	46.10	12.41	+ 35.72	78.20	51.60	45.90	58.57	17.23	+ 11.77
23	63.20	61	65.20	63.13	2.10	+ 85.87	82.20	80	82.20	81.47	1.27	+ 55.47
24	59.40	58	54.70	57.37	2.41	+ 68.89	79.20	81.30	71	77.17	5.44	+ 47.26
25	52.90	72	50.80	58.57	11.69	+ 72.42	71.90	92.90	69.60	78.13	12.84	+ 49.11
26	65.4	66	69.10	66.83	1.98	+ 96.76	The machine reaches its max limit				-	> 69.64
27	61.70	55.80	39.60	52.37	11.44	+ 54.17	75.60	72.50	55.40	67.83	10.88	+ 29.45
28	54.90	41.90	49.20	48.67	6.52	+ 43.28	68.90	52.10	66.90	62.97	9.19	+ 19.53
29	64.80	83.80	64.50	71.03	11.06	+ 109.13	The machine reaches its max limit				-	> 69.64

**Fig. 10:**
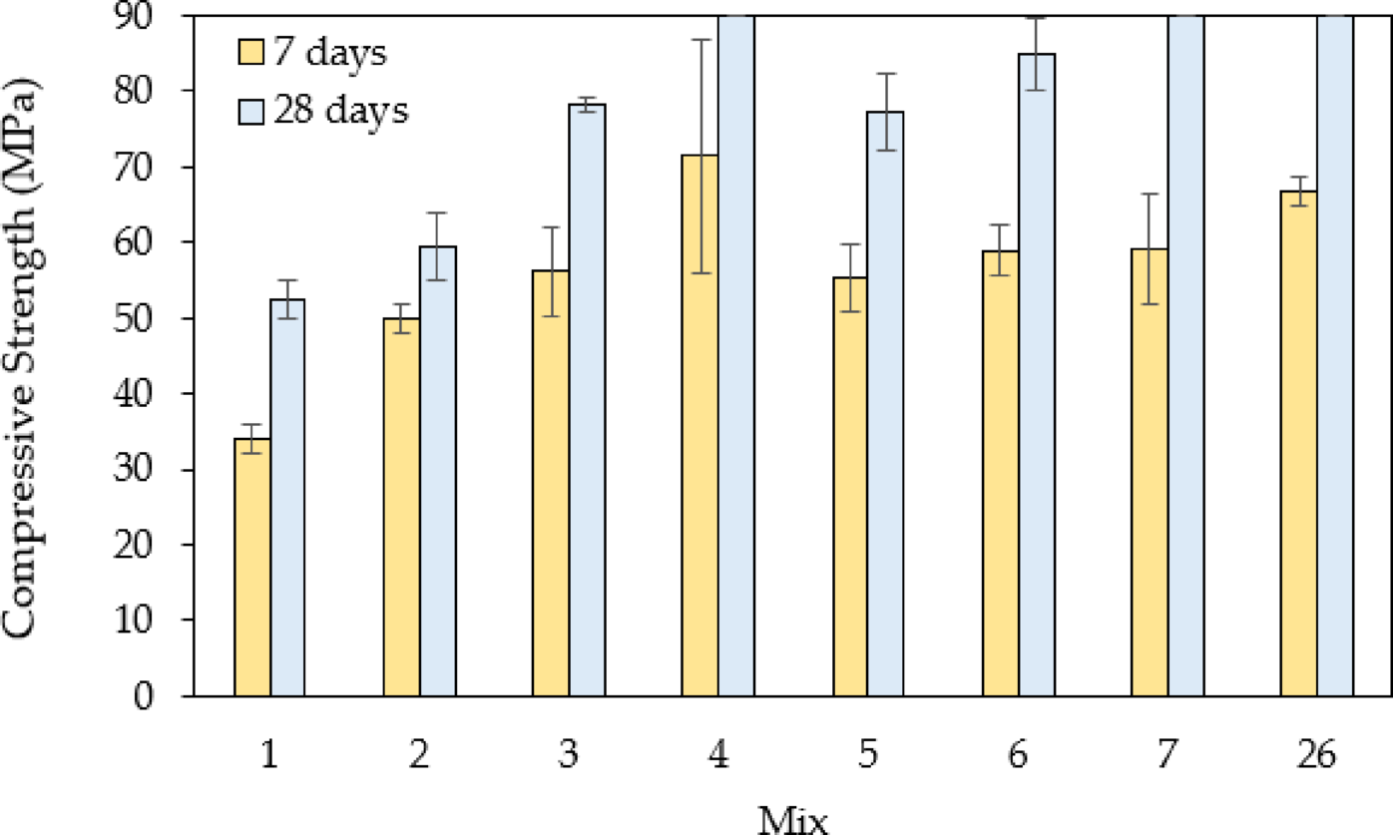
Comparison of compressive strengths for the control mix and mixes with steel slags (full aggregate replacement; without fibers).

The compressive strengths of the first group of concrete mixes incorporating steel slag as a complete replacement for natural aggregates are illustrated in Fig. 10. The results highlight the significant impact of particle size distribution on compressive strength. Mixes 2, 3, and 4, which contain only a single size fraction of steel slag aggregates (either coarse retained on 9.5 mm, coarse retained on 4.75 mm, or fine passing through 4.75 mm, respectively), exhibit relatively high strength values, with improvements of 13.42%, 49.43%, and exceeding 69%, respectively, compared to the control mix. However, despite their strength, the absence of a well-graded aggregate structure can lead to poor particle packing, increased voids, and reduced interlocking which are critical factors for achieving optimal concrete performance. In contrast, Mixes 5, 6, 7, and 26, which incorporate multiple steel slag sizes, demonstrate higher overall compressive strengths, showing increases of 47.52%, 62.53%, exceeding 69%, and 71.25%, respectively. This improvement can be attributed to enhanced packing density and better interparticle interaction, reducing void content and improving load transfer efficiency. The well-graded mixes provide a denser internal structure, leading to higher strength development. Therefore, the results reveal that while individual size fractions of steel slag can achieve relatively high strength, an optimized particle size distribution that includes a blend of fine and coarse aggregates is crucial for maximizing compressive strength and overall concrete performance.

**Fig. 11:**
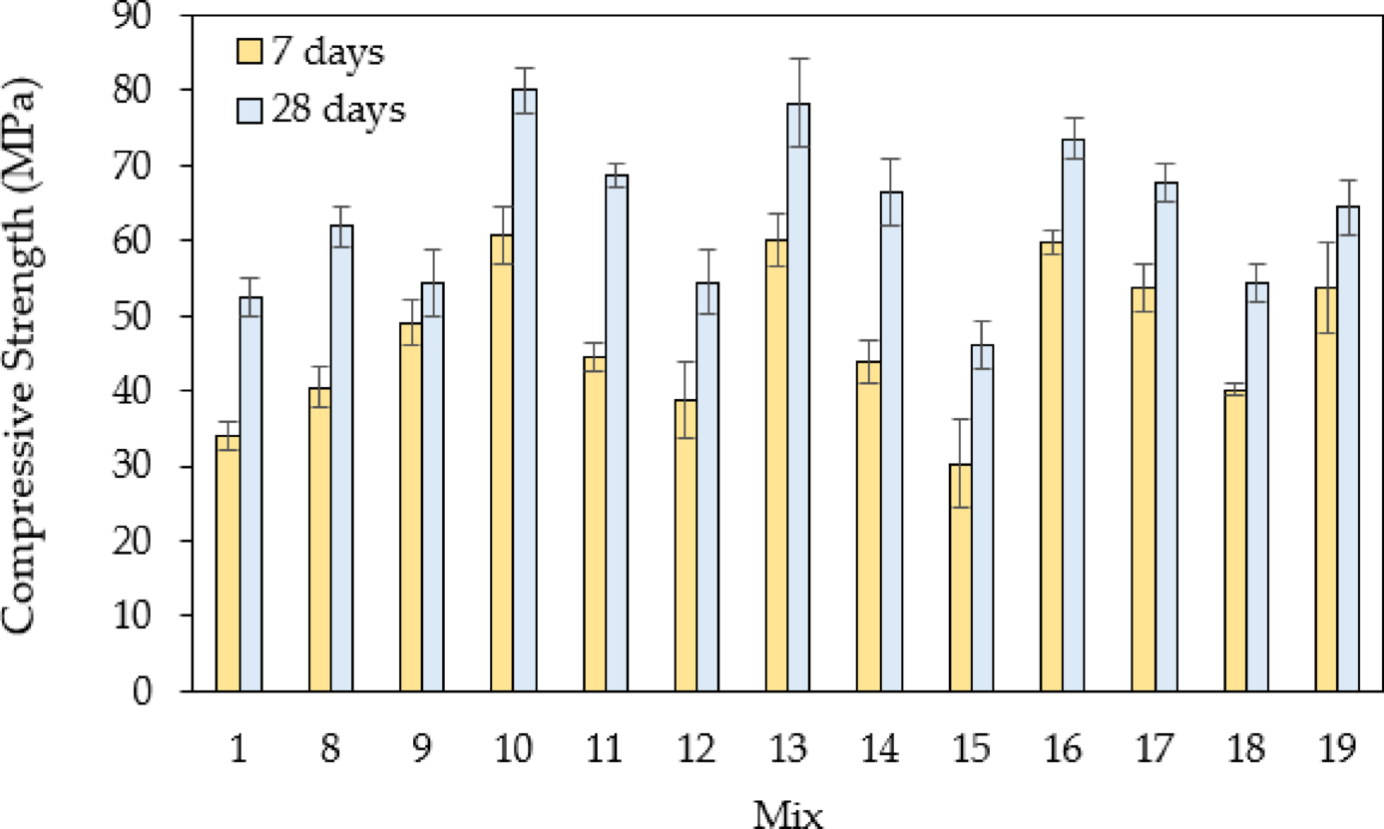
Comparison of compressive strengths for the control mix and mixes with steel slags (partial aggregate replacement; without fibers).

For the second group of concrete mixes incorporating partial replacements of fine and coarse aggregates with steel slag, the compressive strengths are illustrated in Fig. 11. The results demonstrate the influence of different proportions and particle size distributions on compressive strength. Mixes 8 and 9, where 50% of the fine aggregate was replaced with steel slag (passing or retained on 4.75 mm), exhibit compressive strength increases of approximately 10–15% compared to the control mix (Mix 1). This suggests that fine steel slag contributes to strength development, likely due to its angular shape, which enhances interlocking and densification. Mixes 10 to 13 further explore the effect of partial fine aggregate replacement. As the proportion of fine steel slag increases, compressive strength improves. Mix 11, with 50% fine steel slag replacement, shows an increase of 18%, while Mix 13, where 50% of the coarse aggregate was replaced with steel slag retained on 4.75 mm, achieves an increase of 22%. These improvements highlight the beneficial effect of the rough surface texture of steel slag particles in enhancing mechanical interlock. Mixes 14 to 16 focus on coarse aggregate replacement with steel slag retained on 9.5 mm. The trend indicates that increasing the proportion of steel slag leads to higher compressive strengths. Mix 14, with 100% coarse steel slag (9.5 mm) replacement, exhibits a 25% increase in strength, while Mix 16, with 66% replacement, achieves the highest increase of 28%. This can be attributed to enhanced load transfer and the dense packing of the slag particles, which reduce voids. Mixes 17 to 19 replace different percentages of coarse aggregates with steel slag retained on 4.75 mm. The results suggest a similar trend, with Mix 17 (100% replacement) showing a 23% increase and Mix 18 (50% replacement) achieving 19% higher strength. However, excessive use of a single-size fraction, as seen in Mix 19 (75% replacement), leads to a slightly lower increase of 20%, likely due to packing inefficiencies.

**Fig. 12:**
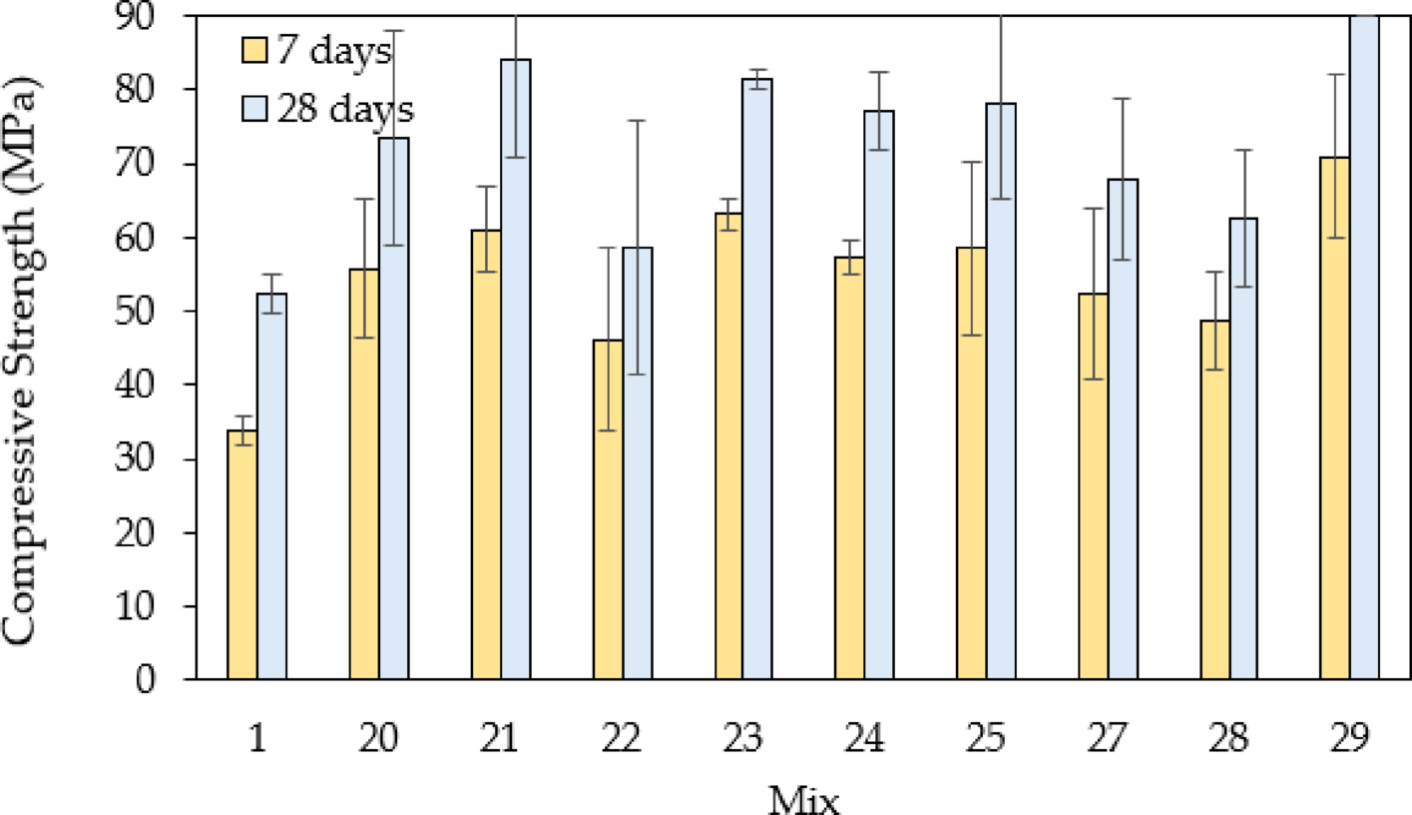
Comparison of compressive strengths for the control mix and mixes with steel slags (full aggregate replacement; with fibers).

Figure 12 illustrates the average compressive strengths for group three concrete mixes with full aggregate replacement of steel slags containing various types of fibers. The results indicate that all modified mixes exhibited higher compressive strengths than the control mix (Mix 1), demonstrating the positive effect of steel slag aggregate and fiber reinforcement on concrete performance. The differences in compressive strengths can be attributed to the variations in particle size distribution, fiber type, and their respective proportions. Mix 29 achieved the highest compressive strength, exceeding 88.89 MPa, which represents an 81.1% increase compared to the control mix. This superior performance is due to the optimized combination of fine and coarse steel slag aggregates, leading to improved particle packing and reduced voids, as well as the presence of macro fibers, which significantly enhance load transfer and crack resistance. On the other hand, Mix 22 had the lowest compressive strength among the modified mixes at 58.57 MPa, only 11.77% higher than the control mix. The relatively lower strength increase can be attributed to the limited amount of polypropylene fibers (0.9 kg), which provide minimal bridging effects and do not significantly contribute to strength enhancement. Additionally, the absence of coarse steel slag retained on the 9.5 mm sieve may have resulted in inadequate interlocking and reduced packing density. Mixes containing steel fibers, such as Mixes 21, 23, and 25, demonstrated significant strength improvements, with Mix 23 reaching 78.45 MPa (a 57.4% increase) and Mix 25 achieving 80.32 MPa (a 60.1% increase). The addition of steel fiber enhanced the tensile strength and ductility of the concrete, improving crack resistance and overall load distribution. Similarly, Mixes incorporating macro fibers, such as Mix 27, also resulted in higher strength.

The influence of silica fumes and superplasticizers was significant across all mixes. The presence of silica fume contributed to enhanced pozzolanic activity, leading to a denser microstructure, while the superplasticizer improves workability and facilitates better particle dispersion. Overall, the findings highlight that an optimized particle size distribution, along with the strategic use of fiber reinforcement, plays a crucial role in maximizing the compressive strength of steel slag-based concrete. The combination of coarse and fine steel slag aggregates, along with macro or steel fibers, was effective in achieving superior strength performance.

It should be mentioned that the observations reported in the current study is further supported by Van Ho Q, Huynh T^9^, who demonstrated a 20–21% unit weight increase in high-performance concrete using 100% steel slag, and by Mu et al.^10^, who reported the formation of dense, C–(A)–S–H-rich microstructures in ultra-high performance concrete incorporating steel slag. Moreover, Le et al.^14^ highlighted the role of optimized filler distribution in achieving high self-sensing performance in UHPC, while Afroughsabet et al.^13^ emphasized the significant mechanical improvements attained when recycled aggregates were combined with steel fibers and slag.

To further validate the improvements in compressive strength observed with the use of steel slag, we conducted statistical analyses on both the 7-day and 28-day data. Two-sample t-tests (assuming unequal variances) were performed to compare each steel slag mix with the control mix at a significance level of α = 0.05. This analysis enabled us to assess the statistical significance of the differences in compressive strength, thereby providing robust support for the beneficial impact of steel slag on concrete performance. Tables 11 and 12 list the 7-day and 28-day compressive strength statistical comparison (control vs. steel slag mixes), respectively. For the 7-day data, the analysis revealed that most steel slag mixes exhibited significantly higher compressive strengths compared to the control mix (*p* < 0.05). For example, Mix 2 (mean = 49.90 MPa) had a t-test p-value < 0.001 compared to Mix 1 (mean = 34.30 MPa), indicating a statistically significant improvement. Similarly, for the 28-day data, the majority of the steel slag mixes showed significant increases in compressive strength relative to the control. Similarly, Mix 24 (mean = 77.17 MPa) demonstrated a highly significant difference (*p* < 0.001) compared to Mix 1 (mean = 52.40 MPa). These statistical results confirm that incorporating steel slag leads to significant enhancements in compressive strength over conventional concrete using natural aggregates.

**Table 11:** 7-Day compressive strength statistical comparison (Control vs. Steel slag Mixes).

Mix	Mean (MPa)	SD (MPa)	t-Statistic	*p*-value
2	49.9	2.02	9.51	< 0.001
3	56.17	5.8	6.18	< 0.001
4	71.43	15.33	4.16	~ 0.03
5	55.4	4.39	7.59	< 0.001
6	58.93	3.35	10.95	< 0.001
7	59.13	7.33	5.66	< 0.01
8	40.43	2.75	3.13	< 0.05
9	49.07	2.92	7.24	< 0.001
10	60.7	3.83	10.6	< 0.001
11	44.53	2.05	6.2	< 0.01
12	38.8	5.15	1.41	> 0.1
13	60.03	3.5	11.05	< 0.001
14	43.9	2.91	4.73	< 0.05
15	30.27	5.9	1.12	> 0.1
16	59.87	1.6	17.4	< 0.001
17	53.8	3.21	8.94	< 0.01
18	40.2	0.8	4.76	< 0.05
19	53.77	5.98	5.35	< 0.01
20	55.8	9.39	3.88	< 0.05
21	61.07	5.74	7.63	< 0.001
22	46.1	12.42	1.62	> 0.1
23	63.13	2.1	17.27	< 0.001
24	57.37	2.41	12.75	< 0.001
25	58.57	11.68	3.55	< 0.05
26	66.83	1.98	20.09	< 0.001
27	52.37	11.44	2.69	< 0.05
28	48.67	6.52	3.66	< 0.05
29	71.03	11.06	5.66	< 0.001

**Table 12:** 28-Day compressive strength statistical comparison (control vs. steel slag mixes).

Mix	Mean (MPa)	SD (MPa)	t-Statistic	*p*-value
2	59.43	4.58	2.3	~ 0.05
3	78.63	0.94	14.73	< 0.001
4	—	—	—	—
5	77.3	4.98	7.66	< 0.001
6	85.17	4.77	10.44	< 0.001
7	—	—	—	—
8	61.87	2.65	4.4	< 0.01
9	54.37	4.49	0.66	> 0.1
10	80.07	3.05	11.92	< 0.001
11	68.67	1.55	9.25	< 0.001
12	54.5	4.35	0.72	> 0.1
13	78.33	5.85	7.01	< 0.001
14	66.4	4.41	4.73	< 0.01
15	46.1	3.21	2.64	< 0.05
16	73.6	2.78	9.64	< 0.001
17	67.8	2.6	7.23	< 0.001
18	54.4	2.67	0.93	> 0.1
19	64.5	3.63	4.69	< 0.01
20	73.63	14.51	2.49	< 0.05
21	84.17	13.16	4.1	< 0.01
22	58.57	17.24	0.61	> 0.1
23	81.47	1.27	17.3	< 0.001
24	77.17	5.44	7.1	< 0.001
25	78.13	12.84	3.4	< 0.05
26	—	—	—	—
27	67.83	10.88	2.39	< 0.05
28	62.97	9.19	1.92	~ 0.10
29	—	—	—	—

In addition to studying the effect of steel slag on concrete compressive strength, this investigation also evaluated the dimensional stability of the specimens over time. Table 13 tabulates the absolute and relative changes in length for different concrete specimens recorded after 7, 14, and 28 days. Although some variations in length were observed, all recorded changes remained below 1%, indicating minimal dimensional instability.

**Table 13:** Change in length for different specimens.

Mixes	Sample Number	Readings (mm)			Change in length (%)		
		7 days	14 days	28 days	7 days	14 days	28 days
1	1	−0.593	+ 0.469	−1.121	+ 0.188	+ 0.612	−0.024
	2	−1.310	+ 1.194	−0.471	−0.004	+ 0.997	+ 0.331
6	3	−0.931	+ 0.912	−0.723	−0.012	+ 0.725	+ 0.071
	4	+ 0.194	−0.235	−1.935	−0.016	−0.187	−0.868
7	5	−0.097	−0.081	−1.745	−0.005	+ 0.002	+ 0.664
	6	−1.169	−0.966	−1.012	+ 0.016	+ 0.097	+ 0.079
11	7	−1.121	+ 1.239	−0.652	−0.008	+ 0.936	+ 0.180
	8	−0.918	+ 1.044	−0.841	+ 0.009	+ 0.794	+ 0.040

### Atomic and molecular structure properties

**Fig. 13:**
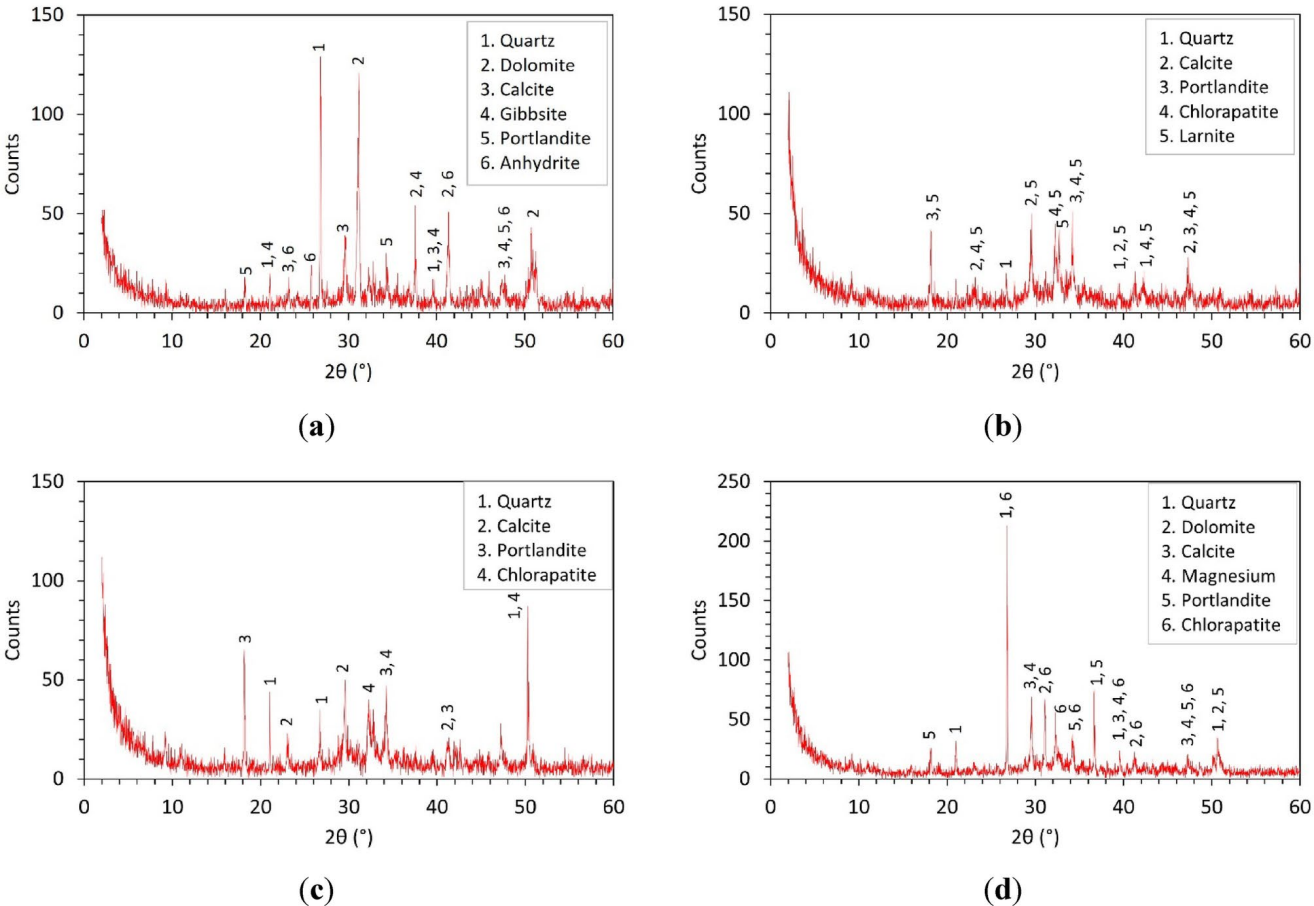
Mineralogical composition of concrete determined by XRD for different specimens: (**a**) mix 1; (**b**) mix 6; (**c**) mix 7; (**d**) mix 11.

XRD analyses were performed on four concrete mixes: Mix 1, Mix 6, Mix 7, and Mix 11 to identify their mineralogical composition, as illustrated in Fig. 13, and correlate it with the observed mechanical performance. The main crystalline phases detected include quartz (SiO₂), dolomite [CaMg(CO₃)₂], calcite (CaCO₃), portlandite [Ca(OH)₂], anhydrite (CaSO₄), larnite (Ca₂SiO₄), chlorapatite [Ca₅(PO₄)₃Cl], and minor phases such as gibbsite [Al(OH)₃]. Peak identification revealed that quartz typically shows strong reflections near 2θ ≈ 20–27°, dolomite and calcite produce prominent peaks at 2θ ≈ 30–31° and 29–30°, respectively, portlandite has characteristic peaks around 2θ ≈ 18°, 34°, and 50°, anhydrite appears near 2θ ≈ 25.5°, 31°, and 50°, larnite is identified near 2θ ≈ 32°, 41°, and 51°, and chlorapatite near 2θ ≈ 26–27°, 32°, and 49–50°. Mix 1 (control) contains dolomite and calcite from natural gravel, whereas Mixes 6 and 7 fully replaced with steel slag exhibit dominant larnite and chlorapatite peaks. Mix 11, which partially replaces natural aggregates with fine slag, shows both carbonate phases and slag-derived silicates. These mineralogical variations correlate with the differences in compressive strength: larnite in slag hydrates to form additional C-S-H, enhancing late-age strength, while dolomite and calcite act primarily as fillers. The portlandite observed across all mixes supports further pozzolanic reactions with slag or silica fume, refining pores and boosting strength. Consequently, slag-rich mixes achieve superior compressive strengths through the synergy of reactive silicate phases, carbonate filler effects, and silica fume-driven pozzolanic activity.

**Fig. 14:**
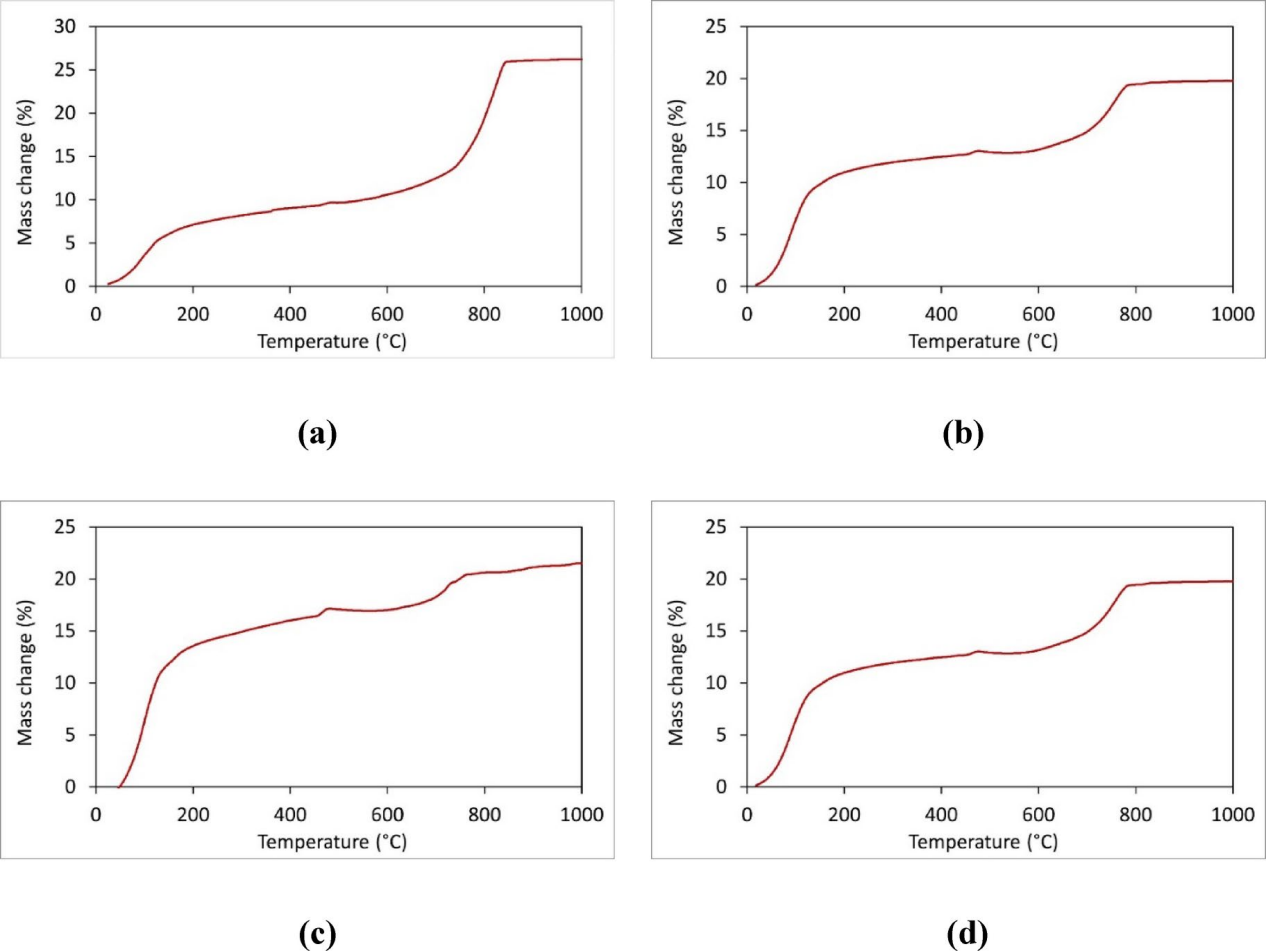
TG-DTA for different specimens: (**a**) mix 1; (**b**) mix 2; (**c**) mix 7; (**d**) mix 11.

The TG-DTA results (Fig. 14) reveal thermal behaviors that correlate well with the observed compressive strengths and the different mixed proportions. In all mixes, the initial mass reduction from 0 to 200 °C corresponds to the evaporation of free and physically bound water, while the gradual mass loss from 200 to 700 °C is associated with the decomposition of hydrated phases like C-S-H and Ca(OH)₂. The results reveal that the mixes incorporating steel slag (Mixes 6, 7, and 11) exhibit a significant mass increase (~ 25%) in the 800–900 °C range, attributed to the reabsorption of moisture by free CaO and MgO present in the slag; this reaction likely promotes further hydration and additional C-S-H formation, leading to a denser microstructure and enhanced compressive strength. In contrast, the control mix (Mix 1) which uses natural aggregates shows the highest overall mass reduction (26.25%), indicative of a less stable hydration system and more porous matrix, which correlates with its lower compressive strength. Thus, the TG-DTA analysis demonstrates that the steel slag’s unique thermal responses, particularly the rehydration of free oxides and the formation of stable oxides and silicates above 900 °C, play a crucial role in improving the microstructural integrity and compressive strength of the concrete.

### Scalability and industrial implications of steel slag in concrete

Steel slag is a widely available by-product of steel manufacturing, making it a promising material for large-scale concrete production. However, its scalability hinges on addressing challenges related to quality variability, proper pre-treatment or stabilization, and logistical factors such as transportation and storage. With robust quality control measures and efficient supply chain management, the integration of steel slag into mass concrete production is feasible and can offer significant environmental and economic benefits.

## Future scope and recommendations

Following the examination of the concrete mix proportions, test outcomes, and associated compressive strength data, the subsequent recommendations are proposed:

- Employ a well-graded combination of steel slag sizes (both fine and coarse) rather than relying on a single-size fraction. Blending these sizes enhances particle packing, reduces voids, and improves interparticle interlocking, which maximizes compressive strength.
- Although full replacement of natural aggregates with steel slag can significantly enhance compressive strength, partial replacement strategies may also offer benefits by balancing cost, workability, and long-term performance. Select the replacement level based on the desired strength and durability criteria.
- The addition of fibers, especially macro and steel fibers, further improves mechanical properties by enhancing toughness and crack resistance. Fiber type and dosage should be optimized based on the overall mix design and target performance.
- Guidelines recommend pre-treating steel slag and adjusting mix design and curing practices for extreme heat to control rapid hydration and moisture loss, while in humid regions, controlling slag reactivity and ensuring proper curing are key to preventing leaching and enhancing durability. National and international standards (e.g., ASTM) provide detailed protocols for its effective use under varying climatic conditions.

Subsequent future investigations are required to investigate the following aspects:

- Investigate the long-term behavior of steel slag concrete, including freeze-thaw resistance, chloride penetration, and carbonation, exposure to ocean water and hazardous chemicals, and high-temperature scenarios.
- Analyze the interaction between steel slag aggregates and reinforced steel to assess potential impacts on bond strength and durability.
- Evaluate various types of steel slag (e.g., ladle slag, BOF slag, electric arc furnace slag) to optimize mix design and performance for diverse applications.
- Examine the behavior of steel slag aggregate concrete under destructive conditions, focusing on its fire resistance and overall structural performance.
- Steel slag in concrete can pose challenges such as heavy metal leaching, expansion and cracking from free CaO and MgO, and inconsistent performance due to material variability. Consequently, further research is needed to develop effective mitigation strategies for these issues.
- Include detailed investigations on how macro fibers affect the cracking behavior and post-cracking ductility of steel slag concrete, incorporating load-deflection, toughness tests, and microstructural analyses to elucidate fiber pull-out mechanisms and residual strength.

## Conclusions

The experimental investigation in this paper demonstrates the potential of steel slag as a sustainable alternative to natural aggregates in concrete production. By developing and evaluating twenty-eight different concrete mix designs, ranging from full to partial replacement of natural aggregates and incorporating various fiber types, we have shown that steel slag can significantly enhance the compressive strength and durability of concrete. The present study, supported by microstructural (TG-DTA and XRD) and statistical evaluations, demonstrates the environmental and technical benefits of utilizing steel slag. The key findings are summarized below:

1. Strength Improvements: G1-Full Replacement of Natural Aggregates with Steel Slag:

- Mixes using a single steel slag size fraction (Mixes 2, 3, and 4) showed compressive strength increases of 13.42%, 49.43%, and over 69%, respectively, compared to the control mix.
- Mixes combining multiple steel slag sizes (Mixes 5, 6, 7, and 26) demonstrated higher compressive strengths, with increases of 47.52% and 62.53% for mixes 5 and 6, respectively, and over 69% for both mixes 7 and 26, attributed to enhanced packing density and improved load transfer.

2. Strength Improvements: G2 -Partial Replacement of Natural Aggregates with Steel Slag:

- Mixes 8 and 9, where 50% of the fine aggregate was replaced with steel slag, achieved a 10–15% increase in compressive strength over the control, suggesting that fine steel slag enhances interlocking and densification.
- Mixes exploring varying proportions revealed that increasing the percentage of fine steel slag (Mix 11) resulted in an 18% increase, while a 50% replacement of coarse aggregates with steel slag (Mix 13) led to a 22% increase.
- For mixes focused on coarse aggregate replacement, 100% replacement (Mix 14) resulted in a 25% increase, and a 66% replacement (Mix 16) achieved the highest gain of 28%.
- Excessive use of a single-size fraction (Mix 19, 75% replacement) slightly reduced the strength improvement to 20%, likely due to packing inefficiencies.

3. Strength Improvements: G3-Steel Slag Concrete with Fiber Reinforcement:

- All fiber-reinforced mixes showed higher compressive strengths than the control, highlighting the synergistic effects of steel slag aggregates and fiber reinforcement.
- Mix 29, which incorporated an optimized combination of fine and coarse steel slag aggregates with 5 kg of macro fibers, achieved the highest compressive strength, representing a 70% increase over the control mix.
- Mix 22, which used a limited amount (0.9 kg) of polypropylene fibers and lacked coarse steel slag retained on a 9.5 mm sieve, exhibited the lowest improvement (11.77% increase).
- Mixes with steel fibers (e.g., Mixes 21, 23, and 25) demonstrated significant enhancements, with increases of 57.4% and 60.1% reported for some mixes.

4. Microstructural Improvements:

- TG-DTA and XRD analyses revealed enhanced hydration and the formation of additional calcium silicate hydrate, resulting in a denser, more stable microstructure in steel slag concrete.

5. Statistical Analysis Validation:

- Comprehensive statistical analyses, including two-sample t-tests, confirmed that the compressive strength improvements observed in the steel slag mixes are statistically significant (*p* < 0.05).
- Graphical error bars further illustrated the low variability and robustness of the performance enhancements.

6. Mixes incorporating fine steel slag aggregates showed the highest compressive strengths, with Mix 4 (using fine slag passing through a 4.75 mm sieve) and Mix 29 (an optimized combination of fine and coarse slag with fiber reinforcement) both exhibiting improvements exceeding 69% compared to the control mix. These performance gains are primarily attributed to enhanced particle packing and additional hydration effects.

While the present study demonstrates promising results and advances our technical understanding of steel slag’s role in enhancing concrete, several limitations must be acknowledged. First, The steel slag used was sourced from a specific production process and may not represent all available types. Second, the sample size for each mix design was relatively small, which may affect the generalizability of the results. Moreover, certain assumptions regarding mix design parameters and curing conditions were made, potentially influencing long-term performance and durability. Despite these constraints, this comprehensive approach contributes significantly to the broader objective of developing sustainable, high-performance construction materials aligned with circular economy principles.

## Data Availability

Data is available from the corresponding author upon reasonable request.
